# Developing a Risk Stratification Model Based on Machine Learning for Targeted Screening of Diabetic Retinopathy in the Indian Population

**DOI:** 10.7759/cureus.45853

**Published:** 2023-09-24

**Authors:** Janani Surya, Himanshu Kashyap, Ramya R Nadig, Rajiv Raman

**Affiliations:** 1 Epidemiology and Biostatistics, National Institute of Epidemiology, Chennai, IND; 2 Shri Bhagwan Mahavir Vitreoretinal Services, Medical Research Foundation, Sankara Nethralaya, Chennai, IND

**Keywords:** random forest, diabetic retinopathy, targeted screening, machine learning, risk stratification

## Abstract

Objective: This study aimed to develop a predictive risk score model based on deep learning (DL) independent of fundus photography, totally reliant on systemic data through targeted screening from a population-based study to diagnose diabetic retinopathy (DR) in the Indian population.

Methods: It involved machine learning application on datasets of a cross-sectional population-based study. A total of 1425 subjects (1175 subjects with known diabetes and 250 with newly diagnosed diabetes) were included in the study. We applied five machine learning algorithms, random forest (RF), logistic regression (LR), support vector machines (SVM), artificial neural networks (ANN), and decision trees (DT), to predict diabetic retinopathy in our datasets. We incorporated a percentage split in the first experiment and randomly divided our data set into 80% as a training set and 20% as a test set. We performed a three-way data split in the second experiment to prevent overestimating predictive performance. We randomly divided our data set into 60% as a training set, 20% as a validation set, and 20% as the test set. Furthermore, we integrated five-fold cross-validation to split the percentage to evaluate our method. We judged the predictive performance based on the receiver operating characteristic (ROC) curve, the area under the curve (AUC), accuracy (Acc), sensitivity, and specificity.

Results: The RF classifier achieved the best prediction performance with AUC, Acc, and sensitivity values of 0.91, 0.89, and 0.90, respectively, in the percentage split. Similarly, a three-way data split attained an outcome of 0.86 and 0.85 in AUC and Acc. Likewise, the five-fold cross-validation performed the best with results of 0.90, 0.97, 0.91, and 0.75 in AUC, Acc, sensitivity, and specificity, respectively.

Conclusion: Since the RF classifier achieved the best performance, we propose it to identify diabetic retinopathy for targeted screening in the general population.

## Introduction

The diabetes mellitus (DM) epidemic is a significant public health concern. With the increasing diabetes trend worldwide, morbidity, mortality, and associated costs of diabetes-related complications are a global public health concern [1-2]. The prevalence of diabetic retinopathy (DR) in India is 12-22.4% [3]. One of the significant risk factors in many population-based studies worldwide is the long duration of diabetes. Population-based studies have identified several risk factors for sight-threatening DR like poor glycemic control, high blood pressure (BP), and insulin usage [4]. Screening of DR involves grading the retinal images using trained graders/ophthalmologists or using artificial intelligence (AI) algorithms that detect DR or sight-threatening DR. The American Academy of Ophthalmology (AAO) screening guidelines for DR state that single-field fundus photography after mydriasis with interpretation by trained readers can be used to identify patients with DR for referral [5]. However, these require a retinal camera to acquire fundus images.

According to the International Diabetes Federation (IDF), in 2019, India had 77 million people with diabetes [6]. Annual retinal photographs for all of them are desired but cannot be achieved. There is a need to develop a scoring system independent of fundus photography for the high-risk population. There are many diabetes risk scores in different ethnicities like Finnish Diabetes Risk Score (FDRS), German Diabetes Risk Score (GDRS), Indian Diabetes Risk Score (IDRS), and Danish Diabetes Risk Score (DDRS) using different variables [7-10]. Various nations and studies have utilized various diabetes risk scores to identify undiagnosed diabetes. In the Indian community, the IDRS screening score has a sensitivity of 72.5% and a specificity of 60.1% [9]. There are attempts to have similar risk scores for DR too. Cassanova et al. graded fundus photography and systemic data from 3443 Action to Control Cardiovascular Risk in Diabetes (ACCORD)-Eye study participants to estimate random forest (RF) and logistic regression (LR) classifiers. It was found that both types of data were informative when discriminating between participants with DR and without DR [11]. Likewise, Tsao et al. studied Northern Taiwan by extracting data from a private hospital database. They proposed a Support Vector Machine (SVM) as a better algorithm that only analyses the systemic data to make predictions [12].

The current study aimed to develop a predictive risk score model based on deep learning to diagnose DR in the Indian population. We intended to create a model independent of fundus photography, totally reliant on systemic data through targeted screening from population-based data from South India.

## Materials and methods

A population-based study from South India, the Sankara Nethralaya Epidemiology and Molecular Genetics Study (SNDREAMS-II), a four-year follow-up study of a cross-sectional survey of people with diabetes, provided the data to develop the models in this study [13]. The study design and research methodology have been described elsewhere [14]. In the initial cross-sectional study, the computed required sample size was 5830. This estimate was based on the idea that there were 1.3% of people with DR in the general population, with a relative precision of 25%, a dropout rate of 20%, and a design effect of 2. The study was carried out in Chennai, Tamil Nadu, which consists of 10 corporation zones with 155 divisions. A multistage systematic random sampling was conducted in two stages: division selection and subject selection. Computer-generated random numbers were used to choose 10 divisions out of 155. This ensured that one division from each corporate zone was included in the sample. People who were eligible for the study were chosen at random from each division. To meet the goal, 600 people were counted in each division (a total of 5999 in 10 zones). Of the 5999 subjects enumerated in the initial study (2003-2006), a total of 1425 subjects (1175 subjects with known diabetes and 250 with newly diagnosed diabetes, defined as fasting plasma glucose ≥110 mg/dL on two occasions) were followed up in this cohort (2007-2011) and were invited to the base hospital for a comprehensive evaluation, including biochemical investigations. Of the 958 patients who had an assessment in the study, there were patients with no DR (n = 828) and patients with DR (n = 130). Participants provided written informed consent after the study was approved by the Institutional Review Board at the Vision Research Foundation in Chennai, India (approval number 59-2007-P). To address the issue of an imbalanced number of subjects between the two groups, which could lead to a biased result favoring the larger group, 130 subjects were randomly selected from the 828 normal subjects to compare with the group with DR.

Machine learning algorithms used to predict DR

Predicting the presence or absence of DR is a multi-class classification problem. We consolidated RF, LR, SVM classifier, artificial neural networks (ANN), and decision trees (DT) to predict DR. 

1. RF builds a combination network of trees and uses two types of randomness: each tree is grown using a boot-strapped version of the training data. The second level of randomness is calculated when the tree is constructed by selecting a random sample of predictors at each node to determine the optimal split [15].

2. LR is a predictive model used to assess the relationship between the target, categorical data with a nominal or ordinal scale, and the predictor, categorical data with an interval or ratio scale [16].

3. SVM is a computer algorithm that learns by example to assign labels to objects. It was developed to efficiently train linear learning machines in kernel-induced feature spaces by applying the generalization theory of Vapnik and co-workers [17].

4. An ANN is a computational and mathematical model that mimics the structures and operations of biological neural networks, such as the human brain. ANNs are routed by many artificial neurons for calculation and are presented as systems of interconnected neurons that send messages to each other [18].

5. A DT is a hierarchical model that consists of nodes, leaves, and branches. The DTs are built through a logic that identifies means to divide the datasets depending on various scenarios [19].

Statistical analysis

We analyzed the statistical significance of categorical and continuous variables using the chi-square test and t-test. Table 1 displays the counts and percentages of DR and No DR groups for each categorical and continuous variable, as well as the statistical analysis (mean and standard deviation) of DR and No DR groups for each variable. Diastolic blood pressure was not statistically significant in the categorical and continuous variables of characteristics between DR and No DR groups. Other variables demonstrated a significant difference between the DR and No DR groups. This showed that our preliminary statistical analysis could identify risk factors that corresponded well with biomedical understanding. Among the variables, it is compelling to observe that variables like gender, insulin usage, duration of the disease, fasting blood sugar (FBS), glycated hemoglobin (HbA1C) levels, and body mass index (BMI) obtained p-values less than 0.001. This also suggests that the clinical care variables of diabetic patients may serve as significant predictors of DR.

**Table 1:** Association between sociodemographic variables and diabetic retinopathy DR: Diabetic Retinopathy; DM: Diabetes Miletus; SD: Standard Deviation; BP: Blood Pressure; FBS: Fasting Blood Sugar; HbA1C: Glycated Hemoglobin; BMI: Body Mass Index

	DR			No DR		P-value
		Number	Percentage (%)	Number	Percentage (%)	
Gender	Male	87	66.9	70	53.8	P<0.001
	Female	43	33.1	60	46.2	
Family History of Diabetes	Yes	90	69.2	70	53.8	0.008
	No	40	30.8	60	46.2	
Insulin	Yes	32	24.6	8	6.2	P<0.001
	No	98	75.4	122	93.8	
		Mean	SD	Mean	SD	P-value
BP Systolic		142.12	23.05	137.4	19.44	0.003
BP Diastolic		80.98	11.01	82.41	11.14	0.076
Age		56.41	8.65	54.94	9.55	0.021
Duration of DM		9.62	6.75	4.15	5.37	P<0.001
FBS		166.66	63.2	143.9	57.12	P<0.001
HbA1C		9.24	2.3	7.12	2.05	P<0.001
BMI		24.66	4.33	25.01	4.42	P<0.001

## Results

Prediction performance is evaluated by a percentage split

**Figure 1:**
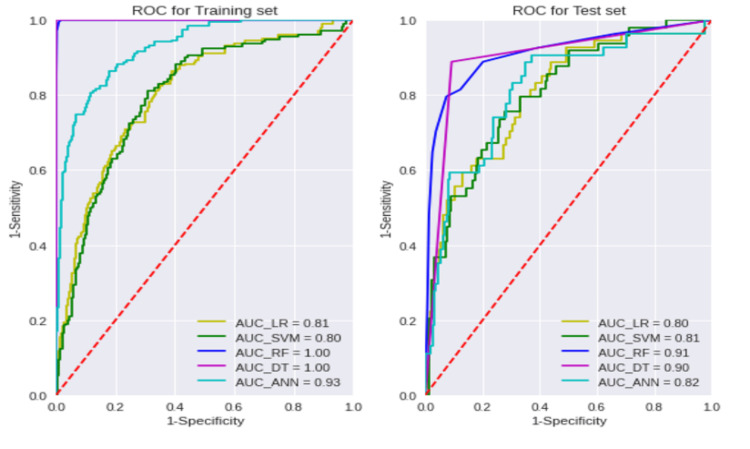
ROC curves of various machine learning algorithms (LR, SVM, RF, DT, and ANN) for the training (80%) and test (20%) data sets. LR: Logistic Regression; SVM: Support Vector Machine; RF: Random Forest; DT: Decision Tree; ANN: Artificial Neural Network; AUC: Area Under the Curve; Receiver Operating Characteristic

To compare with other studies, we incorporated a percentage split in the first experiment and randomly divided our data set into 80% as a training set and 20% as a test set. We applied five machine learning algorithms, as mentioned before, to predict DR, and the predictive performance receiver operating characteristic (ROC) curve is shown in Table 2 and Figure 1, respectively. We used Keras, Numpy, and Scikit learn library in Python version 3.8, a high-level application programming interface (API). Keras is fast, and at the backend, it uses Tensorflow. The area under the curve (AUC) ranked from high to low in the test set were RF, DT, ANN, SVM, and LR. The RF classifier achieved the best prediction performance among the five machine learning algorithms, with AUC, accuracy (Acc), and sensitivity values of 0.91, 0.89, and 0.90, respectively, and DT was second best with performances of 0.86, 0.85, and 0.94 in AUC, Acc, and sensitivity, respectively. This suggests that machine learning algorithms such as RF and DT perform better than the other classifiers for predicting DR. The prediction performance might be exaggerated if the data set was purely divided into two data sets and if the test set is used for parameter calibration and model selection. A three-way data split program was implemented to prevent the overestimation of predictive performance in the subsequent section.

**Table 2:** Prediction performance using percentage split LR: Logistic Regression; SVM: Support Vector Machine; RF: Random Forest; DT: Decision Tree; ANN: Artificial Neural Network; AUC: Area Under the Curve; Acc: Accuracy; Sens.: Sensitivity; Spec.: Specificity

Model	Training				Testing			
	AUC	Acc	Sens.	Spec.	AUC	Acc	Sens.	Spec.
LR	0.81	0.8	0.83	0.64	0.8	0.8	0.81	0.87
SVM	0.8	0.77	0.79	0.56	0.81	0.8	0.83	0.61
RF	1	0.99	0.98	0.99	0.91	0.89	0.9	0.86
DT	1	1	1	0.99	0.86	0.85	0.94	0.66
ANN	0.93	0.88	0.9	0.82	0.82	0.83	0.86	0.68

Prediction performance is evaluated by a three-way data split

**Figure 2:**
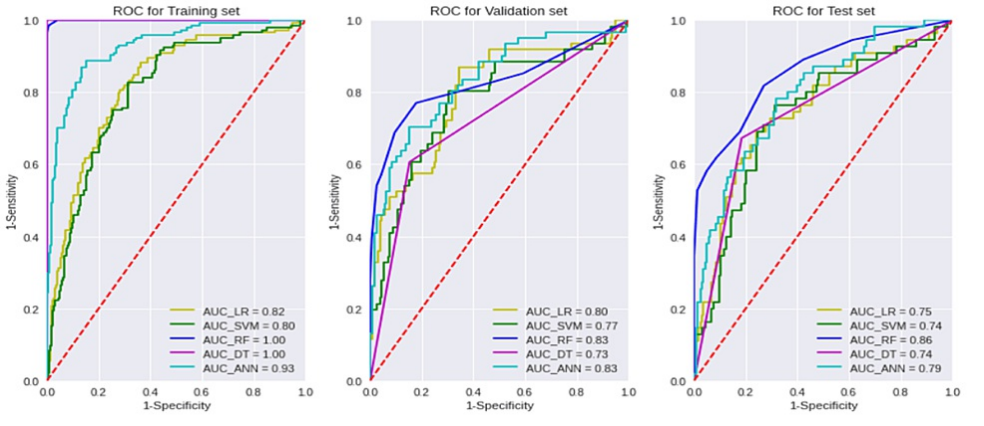
ROC curves of various machine learning algorithms (LR, SVM, RF, DT, and ANN) for the training (60%), validation (20%), and test (20%) data sets. LR: Logistic Regression; SVM: Support Vector Machine; RF: Random Forest; DT: Decision Tree; ANN: Artificial Neural Network; AUC: Area Under the Curve, ROC: Receiver Operating Characteristic

We randomly divided our data set into 60% as a training set, 20% as a validation set, and 20% as a test set. Table 3 and Figure 2 illustrate the three-way data split's prediction performance and ROC plots, respectively. Our RF model achieved 0.85 accuracy and 0.86 AUC when evaluated against an independent test set. In addition, when comparing Table 3 and Table 2, the prediction performance of test sets evaluated by the three-way data split scheme is marginally inferior to that of the percentage split. Therefore, this suggests incorporating a three-way data split method is better for evaluating the actual performance. Compared with other machine learning algorithms, RF obtained the best prediction performance in terms of Acc and AUC in the training, validation, and testing sets. These observations correspond well with our initial percentage split-based experiment.

**Table 3:** Prediction performance using three-way data split LR: Logistic Regression; SVM: Support Vector Machine; RF: Random Forest; DT: Decision Tree; ANN: Artificial Neural Network; AUC: Area Under the Curve; Acc: Accuracy; Sens.: Sensitivity; Spec.: Specificity

Model	Training		Validation		Testing	
	Acc	AUC	Acc	AUC	Acc	AUC
LR	0.8	0.82	0.79	0.8	0.76	0.75
SVM	0.79	0.8	0.75	0.77	0.74	0.74
RF	0.99	1	0.85	0.83	0.85	0.86
DT	1	1	0.78	0.72	0.77	0.73
ANN	0.89	0.93	0.83	0.83	0.79	0.79

Prediction performance using five-fold cross-validation

Furthermore, we integrated five-fold cross-validation to split the percentage to evaluate our method, as shown in Table 4. Randomly dividing our data set into five folds, one fold was selected as the test set while the other four folds were used to train the prediction model. This procedure was repeated five times until each fold had served as the test set. Like our previous tests, RF performed the best with 0.90, 0.97, 0.91, and 0.75 in AUC, Acc, sensitivity, and specificity, respectively.

In addition to the primary variables found to be significant predictors of DR, we also conducted an analysis incorporating lipid metabolism (lipid profiles), diabetic medication usage including oral hypoglycemic agents (OHA) and insulin, as well as the coexistence of micro/macrovascular complications (neuropathy, nephropathy, hypertension, and a history of coronary artery disease (CAD)). Despite their clinical relevance, these additional variables did not demonstrate a statistically significant association with DR and were, therefore, excluded from the final predictive model to maintain its robustness and accuracy.

**Table 4:** Prediction performance using five-fold cross-validation LR: Logistic Regression; SVM: Support Vector Machine; RF: Random Forest; DT: Decision Tree; ANN: Artificial Neural Network; AUC: Area Under the Curve

Model	AUC	Accuracy	Sensitivity	Specificity
LR	0.76	0.8	0.8	0.54
SVM	0.74	0.79	0.78	0.49
RF	0.9	0.97	0.91	0.75
DT	0.85	0.9	0.95	0.56
ANN	0.78	0.86	0.83	0.55

## Discussion

Several previous studies have assimilated machine learning algorithms to predict DR, and the performance of their proposed approaches is summarised in Table 5. Butt et al. employed three different classifiers, i.e., RF, multilayer perceptron (MLP), and LR, for diabetes classification. During the analysis, they observed that RF could achieve an Acc of 0.774 [20]. Sun and Zhang used electronic health records to diagnose and analyze DR. They took the data from the Medical Big Data Center in China, which was taken from 301 hospitals over four years. In this work, they have concluded that the machine learning technique. The RF obtained the highest Acc with 0.92 [21].

Reddy et al. trained various algorithms to train predictive models for DR screening; Comparative analysis showed that SVM using the Gaussian kernel had obtained better metrics, and the performance of the RF classifier was comparable [22]. Gupta et al. used two publicly available datasets, namely, the laser marks dataset for DR screening (LMD-DRS), and the laser marks dataset before and after photocoagulation treatment (LMD-BAPT). RF could achieve Acc of 0.867, sensitivity of 0.875, and specificity of 0.894 for the LMD-DRS dataset and 0.927, 0.889, and 0.958, respectively, for the LMD-BAPT dataset [23]. This supports our hypothesis that incorporating machine learning algorithms with discriminative clinical features could efficaciously detect DR and thus optimise cost-effectiveness in healthcare systems. 

**Table 5:** Random forest algorithm comparison with previous studies AUC: Area Under the Curve; NA: Not Available

Approaches	Data Sets	AUC	Accuracy	Sensitivity	Specificity
Butt et al. [20]	India	NA	0.77	NA	NA
Cassanova et al. [11]	USA	>0.75	NA	NA	NA
Sun and Zhang. [21]	China	NA	0.92	NA	NA
Reddy et al. [22]	India	NA	0.68	0.67	0.7
Gupta et al. [23]	LMD-DRS (India)	NA	0.86	0.87	0.89
Gupta et al. [23]	LMD-BAPT (India)	NA	0.92	0.89	0.95

Since the RF classifier achieved the best performance of 0.91 in AUC, 0.89 in Acc, 0.90 in sensitivity, and 0.86 in specificity, and the performances have been relatively stable and repeatable as compared to other machine learning algorithms in our various tests, we propose it as the method to identify DR for targeted screening in the general population.

Limitations of this study

In India, the prevalence of sight-threatening DR among people with diabetes has increased dramatically. This paper aims to demonstrate the viability of our methods in the field of DR prediction. However, our study has some limitations. Our study was based on a small sample of the population-based study of Chennai, India. In the small group of diabetic patients, duration of disease, higher HbA1C levels, FBS, BMI, and insulin therapy were risk factors. We only provide individual analyses for the small group; hence, the resulting risk factor cannot be applied to the entire Indian population. A primary limitation is that our analyses were conducted using graded fundus photography data instead of the actual images. To implement the approach presented here, classification should be performed automatically using fundus photographs in place of expert grading. Utilizing other types of information, such as genetic data when available, is another potential source of improvement.

## Conclusions

In this study, we undertook a comprehensive approach to identify risk factors for DR, incorporating a wide array of variables ranging from medical histories of both ocular and systemic conditions to anthropometric and biochemical measurements. We utilized machine learning algorithms, notably RF, to predict DR risk based on this extensive dataset. The RF algorithm demonstrated superior performance with an accuracy of 89% and an AUC of 91%, indicating its potential utility in clinical diagnosis and tracking of DR progression. 

Our findings offer valuable insights into the risk factors for DR and demonstrate the efficacy of machine learning techniques, particularly RF, in predicting DR risk. These results have significant implications for the development of clinical decision support systems aimed at early diagnosis and management of DR. As healthcare moves towards data-driven decision-making, our study serves as a foundational step for implementing machine learning algorithms in clinical settings to improve patient outcomes in the foreseeable future.
